# Validity and reliability of the Persian version of modified motivated strategies for learning questionnaire: a methodological study among medical students

**DOI:** 10.1186/s12909-023-04547-z

**Published:** 2023-08-07

**Authors:** Niusha Fakhri, Mitra Amini, Mahsa Moosavi, Erfan Taherifard, Mahboobeh Saber

**Affiliations:** 1 MPH department, Shiraz University of Medical Sciences, Shiraz, Iran; 2 Clinical Education Research Center, Shiraz University of Medical Sciences, Shiraz, Iran; 3 Department of Medical Ethics and Philosophy of Health, School of Medicine, Shiraz University of Medical Sciences, Block No.2, Zand Ave., Imam Hussein Sq, Shiraz, Fars Iran

**Keywords:** Education, medical, continuing, Reproducibility of results, Surveys and questionnaires

## Abstract

**Background:**

Medical undergraduates need to improve their techniques for learning in the different settings of learning in clinical rotations. Reflective learning, in which a person can learn from their experiences, is among the most well-known learning skills. In this study, we aim to translate the newly developed modified form of the motivated strategies for learning questionnaire (MSLQ) to Persian and evaluate its reliability and validity among medical students.

**Methods:**

This study was performed on medical students in clinical stages at the Shiraz University of Medical Science in 2022. The modified MSLQ questionnaire was used in this study which is a 32-item tool measuring different aspects of self-reflecting, including self-orientation, feedback-seeking, critical thinking, and self-regulation. This questionnaire was translated into Persian properly. Cronbach’s alpha and confirmatory factor analysis were used to ascertain the reliability and validity of the tool.

**Results:**

A total of 325 medical students consisting of 174 men and 151 women with an average age of 23.79 (± 2.21) were enrolled. Path diagrams of confirmatory factor analysis for both standardized regression coefficients and t-values and all the fitness indicators were in favor of the proper validation of the translated version. The overall Cronbach’s alpha for the questionnaire was 0.9, and the value for each of four subscales was above 0.7.

**Conclusions:**

Our study showed that the Persian-translated version of the modified MSLQ is valid and reliable without taking too much time and effort to implement. We recommend that the developed tool be distributed to medical students from other Iran universities.

## Background

A successful medical education system should be able to produce clinically skilled physicians who can use their skills and knowledge in the best possible way. Physicians must always integrate their knowledge, skills, and experience to help patients achieve their health goals [1]. Another necessity for becoming a successful physician is engaging in lifelong learning. Knowledge, information, and technology are rapidly changing in the health sector. In such an environment, physicians must keep their knowledge and skills updated to take better care of their patients [2, 3]. This makes lifelong learning crucial for improving patient outcomes [4].

Until now, there is no wholly accepted method for becoming an excellent lifelong learner, but it is agreed that physicians need specific learning skills to become effective. Reflective learning is one of the most well-known learning skills in which people can learn from their experiences and mistakes [5, 6]. In reflective learning, a person reflects on a background and explores the concerns triggered by that experience. This will improve one person’s function in similar situations in the future. The process of reflection is necessary for lifelong learning and achieving success in the medical career. Research shows that there is an excellent chance that individuals reflect as a natural process, but they might not consider it as a learning tool. Making them aware of the process can help them control and use it as a learning tool [7]. Over the past decade, reflective learning and how it can be taught and measured have become a matter of concern. A lot of researchers studied ways to improve reflective learning among students. Others showed its positive effects on diagnosing and managing complex cases [8, 9].

One way to improve reflective learning is by developing instruments to measure it. The reflection-in-learning scale is one of the tools that can be used to measure medical students’ reflective learning; researches show its construct validity, but its main focus is on the cognitive dimension of reflective learning [10]. Another study by Soemantri et al. developed a new instrument for measuring medical students’ reflections on learning. The purpose of this instrument is to measure different aspects of reflective learning, such as cognitive, metacognitive, emotional, and motivational. This study used the old motivated strategies for learning questionnaire (MSLQ) and made some changes to make it suitable for its purpose [11]. MSLQ is a questionnaire with 81 items that assess college students’ motivational learning strategies. This questionnaire consists of two main sections, a motivational section, and a learning strategies section. This questionnaire generally studies three main factors of self-regulated learning, motivation, metacognition, and behavior [12]. Further, other studies showed it could be a reliable questionnaire across various samples, but researchers should always study the reliability of the scores of their sample [13]. All these characteristics make MSLQ a good reference for making an instrument for evaluating reflective learning in medical students.

Considering the critical role of the use of proper techniques for medical education and the importance of reflective learning as a proper and well-known one, it is necessary to explore ways to improve it and help medical students get familiar with it, and use instruments to measure their reflective learning. The purpose of this study is to translate the newly developed modified form of the MSLQ questionnaire to Persian and assess its reliability and validity among medical students. The results of this study could provide medical educators and students with a reliable and valid instrument to assess reflective learning. This, in turn, could help improve medical education and learning by identifying areas where students may need additional support to develop their reflective learning skills and become effective lifelong learners.

## Methods

### Methods and design

This study was a cross-sectional work and was performed on 325 medical students of the Shiraz University of Medical Science, Shiraz, Iran, from January to February 2022. Based on a literature review done by Emmanuelle Anthoine and the fact that this is our first time using this questionnaire in Iran, the proper sample size was determined to be ten times more than the number of questions [14]. This questionnaire has 32 questions, so we decided to enroll 320 medical students. In this study, we only considered medical students on the clinical rotations as some of the questions in this tool are on learning clinical skills. Therefore, students in the preclinical years have not entered the study. The COnsensus-based Standards for the Selection of health status Measurement Instruments (COSMIN) guideline, its terminologies, and its definitions were used for a uniform reporting of measurement properties and to assure the methodological quality of our work [15, 16].

The survey was conducted completely anonymously, and the participants’ identity was not gathered with our data from each participant. We also assured participants that their answers would remain confidential and obtained their consent. All participants were aware of their right to refuse that their data be analyzed in the study.

### Data gathering tool

The modified MSLQ questionnaire is an instrument for measuring medical students’ reflective learning [11]. To make this instrument, 36 questions were selected from the original MSLQ questionnaire. Then the questions underwent some revisions to make them more relevant to medical students. This 36-item questionnaire went under three phases of the research program, and the final result was a 32-item questionnaire, scored on a 7-point Likert scale (from 1 = not at all true of me to 7 = very true of me). This questionnaire contains 4 subscales. These subscales show different dimensions of reflective learning. Self-orientation, feedback-seeking, critical thinking, and self-regulation are the four subscales of this questionnaire.

### Persian translation of the modified MSLQ and assessing its content validity

The modified MSLQ instrument was translated considering the four stages recommended by Chen et al. for the translation procedure [17]. Before starting the research, we explained our work to the author of the modified MSLQ questionnaire through an email and obtained her permission to use the questionnaire. At first, two translators, with Persian mother tongue language and proficient in English, independently translated the original questionnaire into Persian. One of these translators was an expert in the field of medical education and the other was not familiar. Then, they both discussed their translated versions and reached an agreement. Afterward, the translated version was back-translated into English by two native English speakers to guarantee its validity. After this, a comparison was made between the translated English versions and the original questionnaire to verify the preservation of the items and domains’ original meaning following the Forward and backward translation processes. The forward and backward translation procedures were utilized to ensure conceptual equivalence between the two versions. The translated version of the questionnaire, which resulted from the past two steps, was then reviewed by the medical education experts and the researcher team. Minor modifications were made to the Persian version of the questionnaire to ensure that the language and cultural context were appropriate for medical students in Iran and to improve the simplicity and clarity of some items.

After that the expert panels revised the translation and modifications were made, the Persian version of the questionnaire was sent to a group of faculty members in the department of medical education to check its face and content validity by stating a judgment about the relevance and comprehensiveness of the items. To do this, we calculated the content validity ratio (CVR) and content validity index (CVI), which were done in two stages [18–20].

To calculate the CVR, eight professional faculty members with extensive experience in educational research were engaged in the process of assessing the relevance and necessity of each translated item. The experts individually provided judgments for each item based on Lawshe’s method proposed in 1975, using a Likert scale. The scale consisted of three options: “it is necessary”, “it is useful but not necessary”, or “it is not necessary”. Subsequently, the CVR was computed using the formula CVR = (Ne - N/2) / (N/2), where Ne represents the number of experts indicating “it is necessary” for a particular item, and N is the total number of participating experts.

Furthermore, the CVI was evaluated in the second stage of content validity assessment. The same panel of eight expert faculty members rated the relevance of each item using a 4-point Likert scale. The scale included the following response options: “It is not relevant” (scored as 1), “It is relatively relevant” (scored as 2), “It is relevant” (scored as 3), and “It is highly relevant” (scored as 4). The CVI for each item was then calculated as the proportion of experts giving a rating of either “relevant” or “highly relevant” for that item.

The CVR and CVI values for each item were carefully reviewed and discussed by the research team. Any potential incongruities or ambiguities in the questionnaire items were addressed and revised accordingly to ensure the content validity of the final translated version of the modified MSLQ questionnaire. Subsequently, the final validated version of the modified MSLQ questionnaire was distributed among the medical students for further data collection and analysis.

### Statistical analysis

We used International Business Machines Corporation Statistical Package for Social Sciences (IBM SPSS) version 24 (C.A., The United States of America) and Linear Structural Relations (LISREL) version 7.8 for statistical analyses in the study. We calculated Cronbach’s alpha to measure the internal consistency and reliability and all the participants, 325 medical students, contributed to confirm the reliability values. Cronbach’s alpha was calculated for all of the items together and also for each subscale separately. In order to consider the questionnaire reliable, the coefficient alpha should be above 0.7. In order to study factor structure validity, we used confirmatory factor analysis (CFA). To evaluate the goodness of the model, we used some criteria such as Chi-square statistics, root mean square error of approximation (RMSEA), Chi-degree freedom (CMIN/DF), incremental fit index (IFI), relative fit index (RFI), normed fit index (NFI), the goodness of fit (GFI), adjusted goodness of fit (AGFI) and comparative fit index (CFI).

## Results

### Characteristics of the participants

A total of 325 medical students were included in this methodological study (Table 1). Among them, 143 individuals (44%) were interns, 86 (26%) were stagers and the rest were students. There were 174 men and 151 women with an average age of 23.79 (± 2.21) years. The lowest and highest age of the participants were 18 and 34 years, respectively.

**Table 1:** Demographic characteristics of the participants enrolled in the study

Variables		Ferequency	Percentage
Sex	Female	151	46.5
	Male	174	53.5
Clinical stage	Interns	143	44
	Stagers	86	26
	Students	96	30

### Validity

**Fig. 1:**
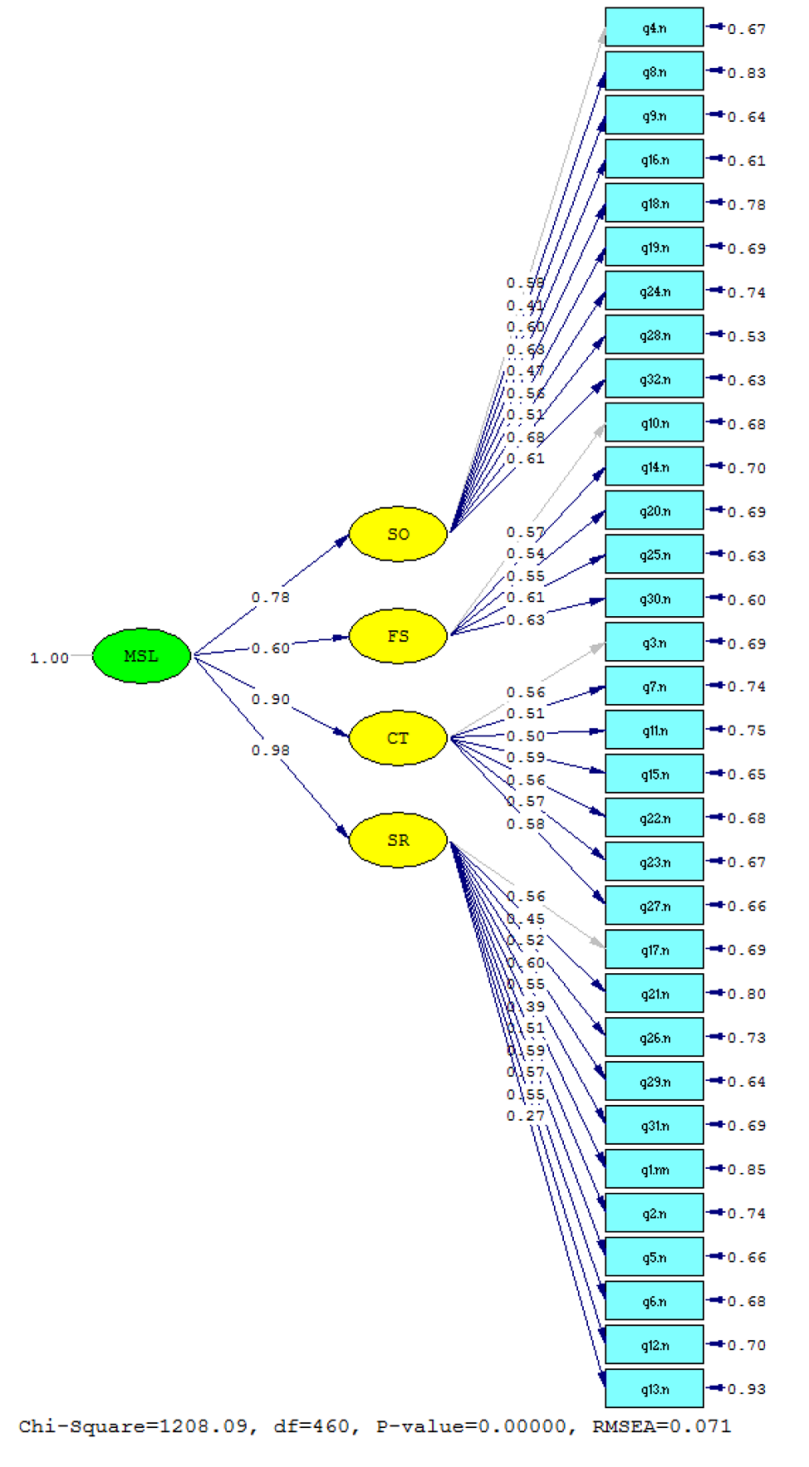
The results of the second-order CFA presented with standardized coefficients

**Fig. 2:**
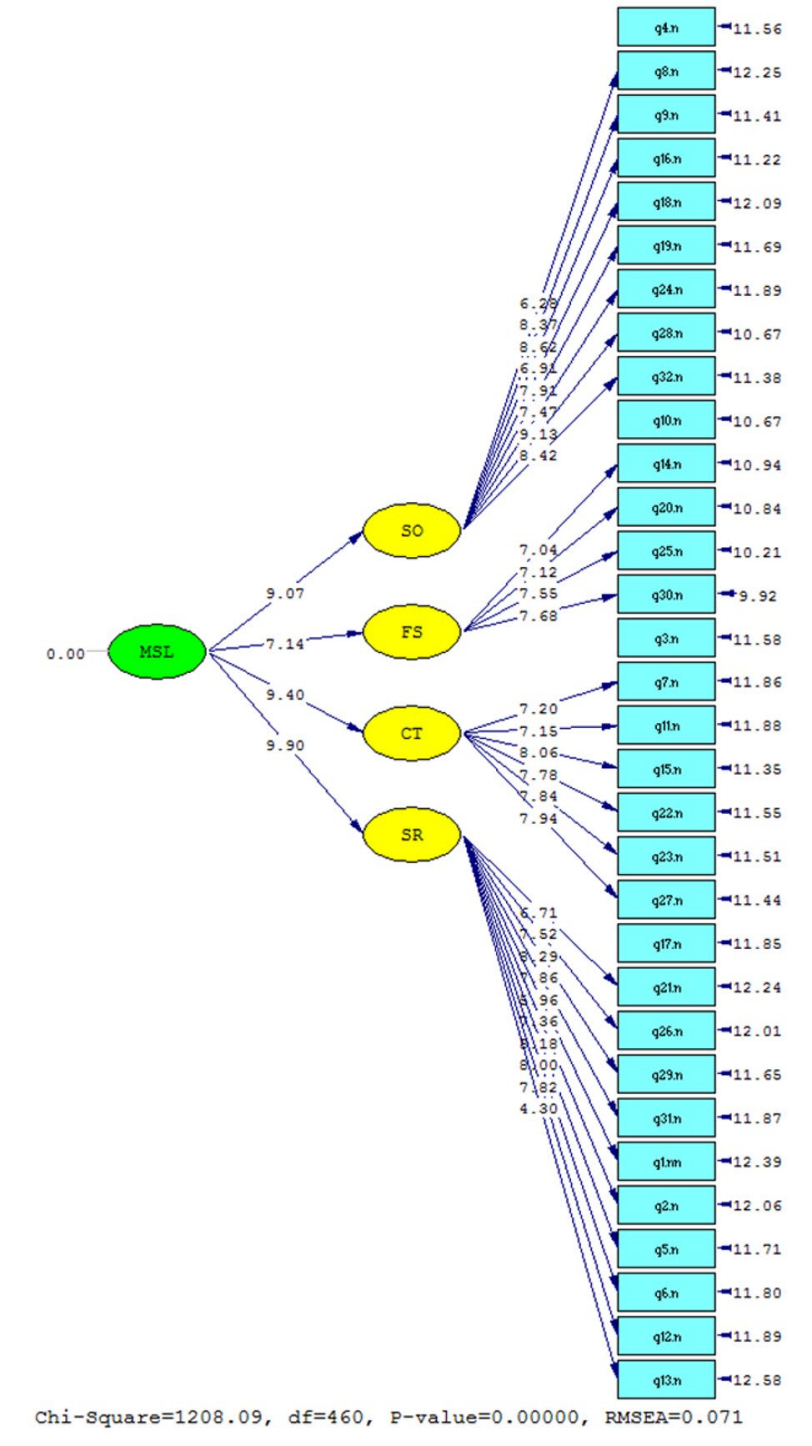
The results of the second-order CFA presented with t-values

The CVI values were equal to or greater than 0.79 for all questions, and the CVR value was 0.75 for the total scale. As shown in Figs. 1 and 2, each item of the questionnaire has a loading corresponding to each of the subscales, which are presented with standardized coefficients and t-values. All the values of the factor loadings are higher than 0.3. CFA also calculated fitness indicators: RMSEA, CMIN/DF, IFI, RFI, NFI, GFI, AGFI, and CFI. These indicators all met the fitness standards (Table 2); therefore, all the statistics confirmed the acceptable fitness of the final model. A path diagram for t-values was also designed in which all the relationships have been demonstrated to be significant.

**Table 2:** The results of the CFA for the proposed model

Fitness indicators	Fit indicators obtained	Threshold
Root Mean Square Error of Approximation	0.071	< 0.1
Chi-Degree Freedom	2.62	< 3
Incremental Fit Index	0.93	>=0.90
Relative Fit Index	0.89	>=0.80
Normed Fit Index	0.89	>=0.80
Goodness of Fit	0.91	>=0.90
Adjusted Goodness of Fit	0.87	>=0.80
Comparative Fit Index	0.93	>=0.90

### Reliability

The results of reliability statistics for different subscales of the questionnaire used in this study are provided in Table 3. The overall Cronbach’s alpha for the questionnaire was 0.9, and the value for each subscale was above 0.7, indicating the tool’s satisfactory internal consistency.

**Table 3:** The reliability coefficients of the Persian version of the modified MSLQ

Subscales	Number of items	Cronbach’s α	Intraclass correlation coefficient	95% Confidence interval	p-value
Self-orientation	9	0.8	0.797	0.762–0.828	< 0.001
Feedback seeking	5	0.721	0.716	0.664–0.762	< 0.001
Critical thinking	7	0.755	0.756	0.713–0.794	< 0.001
Self-regulation	11	0.782	0.786	0.750–0.819	< 0.001

## Discussion

The validity and reliability of this Persian version of the modified MSLQ were assessed in our study after the completion of the questionnaire by 325 medical students in different stages. The results of CFA, fitness indicators, and reliability statistics all confirmed that this 4-factor model used in the study was both valid and reliable. Therefore, the original version of the modified MSLQ could be applied to this Persian translation.

In general terms, the majority results of validity and reliability scores obtained in the analyses of the study were acceptable and quite similar to those previously reported values for the original versions of MSLQ and the modified MSLQ translated in this study; therefore, the results were satisfactory. The MSLQ has been translated by researchers into more than twenty languages and its psychometric properties were assessed and showed satisfactory results [13, 21]. Besides, various modifications have been made to the MSLQ for different settings, contexts, and target groups and these modified versions were also translated to different languages and their validity and reliability were measured. These attempts were also made for translation and evaluation of the MSLQ into Persian for the Iranian context. In a study by Dortaj and Afsharian, 337 high school students from Tehran, Iran, were enrolled, and the psychometric properties of the translated version of MSLQ were assessed [22]. This study found that the overall Cronbach’s alpha of this Persian version and each subscale’s alpha value were above 0.7, ranging from 0.78 to 0.91, which indicates that the translated version of the questionnaire is reliable. Moreover, fitness indicators including CMIN/DF, IFI, GFI, RMSEA, CFI, and AGFI were measured, all showing a great fit of the Persian-translated questionnaire. In another similar methodological study in Iran, high school students were evaluated for their learning strategies using a translated version of the MSLQ [23]. In this study, it was reported that MSLQ had a suitable fit for the population with a GFI of  0.97, AGFI of 0.95, RMSEA of 0.044, and CMIN/DF of 2.10. These studies show that applying MSLQ may be suitable in an Iranian context.

The reliability and validity of the Persian version of MSLQ were also assessed among Iranian undergraduate students of the medical sciences field. In a study of 391 undergraduate participants from schools of health, paramedic, rehabilitation, and nursing and midwifery at Zanjan University of Medical Sciences, a translated version of MSLQ was distributed and it was shown that it had reliability indices comparable to those of non-translated original MSLQ [24], reliability of 0.79, 0.80, 0.82, 0.78 and 0.77 for subscales of self-efficacy, intrinsic value, test anxiety, cognitive strategies use and self-regulation, respectively in comparison to 0.89, 0.87, 0.75, 0.83 and 0.74 value for the original Pintrich et al. MSLQ [25]. It was also stated that the questionnaire was valid. The modified version of MSLQ designed by Soemantri et al. for medical students was, however, not previously translated and our study is the first study that assessed the reliability and validity of this questionnaire in another language [11]. In this research conducted by Soemantri et al., it was found that modified MSLQ for medical students has an excellent internal consistency in all its subscales with an alpha coefficient of 0.87 for self-orientation, 0.73 for feedback seeking, 0.77 for critical thinking, and 0.67 for self-regulation. Cronbach’s alphas for the coefficients of the subscales of self-orientation, feedback seeking, critical thinking, and self-regulation in the Persian format of the questionnaire were 0.8, 0.721, 0.755, and 0.782, respectively, demonstrative of good reliability of the tool [26]. Therefore, the following studies could be conducted with this current questionnaire format.

The value of RMSEA calculated in this study was 0.071. This indicator is among the most commonly used statistics to assess whether the structural modeling proposed has appropriate fitness or not [27, 28]; values below 0.1 show a fair fit, and therefore, the model covariance has no significant difference with covariance matrices of the medical students sampled in our study. Both the GFI and AGFI, the R-squared measure, and the adjusted R-squared measure analogs in the regression analysis modeling are indicators of the extent of existing variance, which could be attributed to the observed population covariance [29]. Both measures are above the cutoff for a good fit, suggesting this four-factor structural model could be used in the current format for medical students. Another indicator was CMIN/DF, which is calculated by dividing chi-square by the degree of freedom, and values below 3 show good model fitness. For our study, the estimated chi-square and degree of freedom were 1208.09 and 460, respectively; therefore, the CMIN/DF was 2.62. Other fit indices were IFI, RFI, NFI, and CFI, which assess the model’s goodness [30], and they all were indicative of a good model fit. Therefore, given these values, it seems that the current format of the model fits the data we gathered from these participants, and none of the indices were out of the accepted ranges. However, more studies are required to optimize this four-factor model for medical students in different universities in Iran. Besides these fit indicators, path diagrams of CFA for both standardized regression coefficients and t-values, the values, the correlations, and the subscales, showed that the gathered data in this study fit the four-factor model.

Although this study had a statistically appropriate sample size and reasonable response rate, there were some limitations in this work. The study was performed in one medical university among undergraduate students, and the method of sampling was convenience sampling; so, the results may not be generalizable to other universities and should be approached with caution.

## Conclusions

It is highly important for healthcare workers, especially medical undergraduates, to improve the techniques they use for learning in the different settings of learning in clinical rotations; therefore, monitoring and assessing their learning should be among the priorities of medical universities. Our study showed that the Persian-translated version of the modified MSLQ is valid and reliable without taking too much time and effort to implement. We recommend that the developed tool be distributed to medical students from other Iran universities.

## Data Availability

The datasets used and/or analyzed during the current study are available from the corresponding author upon reasonable request.

## List of abbreviations

AGFI: Adjusted goodness of fit
CFA: Confirmatory factor analysis
CFI: Comparative fit index
CMIN/DF: Chi-degree freedom
GFI: Goodness of fit
CVI: Content validity index
CVR: Content validity ratio
IBM SPSS: International Business Machines Corporation Statistical Package for Social Sciences
IFI: Incremental fit index
LISREL: Linear Structural Relations
MSLQ: Motivated strategies for learning questionnaire
NFI: Normed fit index
RFI: Relative fit index
RMSEA: Root mean square error of approximation
